# Technical Efficiency of Regional Public Hospitals in China Based on the Three-Stage DEA

**DOI:** 10.3390/ijerph17249383

**Published:** 2020-12-15

**Authors:** Zhensheng Chen, Xueli Chen, Xiaoqing Gan, Kaixuan Bai, Tomas Baležentis, Lixin Cui

**Affiliations:** 1 School of Management, Nanchang University, Nanchang 330031, China; zhensheng_chen@163.com (Z.C.); xgan@ncu.edu.cn (X.G.); 2 Institute of Journalism and Communication, Chinese Academy of Social Sciences, Beijing 100026, China; chenxl@cass.org.cn; 3 School of Management and Economics, Beijing Institute of Technology, Beijing 100081, China; 3120201537@bit.edu.cn; 4 Lithuanian Institute of Agrarian Economics, Vilnius 03220, Lithuania; tomas@laei.lt

**Keywords:** efficiency estimation, public hospitals, three-stage DEA, Social Functions

## Abstract

Many countries are facing the increasing cost of healthcare services and the low efficiency of public hospitals. These issues are also evident in China. This paper offers a comprehensive assessment of the efficiency of public hospitals operating in China’s 31 regions. The impact of the third round of reform of the health system in 2009 is assessed based on the three-stage data envelopment analysis procedure. The time period from 2011 to 2018 is covered in this study. Due to different functions performed by the public hospitals and other ones, the number of patients with infectious diseases is incorporated as an output variable reflecting the social function. The outpatient visits and inpatient visits are considered to reflect the outputs related to the private functions. The results imply an increase in the mean efficiency of public hospitals from 0.927 to 0.981 after taking the impact of environmental variables and statistic noise into account. These results indicate that the efficiency of public hospitals is dependent in the operational environment. There are 11 regions whose hospitals operate on the efficiency frontier during the whole period covered. Therefore, the Chinese government should reasonably design and apply the regulatory tools to improve the efficiency of public hospitals.

## 1. Introduction

It is the increasing cost of health care services delivery that has caused concerns in some countries. China has also faced such challenges. Specifically, China has been experiencing an increase in population, rapid aging of the population and improvement of living standards of residents. All of these factors have led to the increasing demand for health services. In the meantime, China entered the “new normal stage” of the economy in 2014, so it is impossible to increase the supply of health services through building new medical institutions and expanding the operation scale of hospitals as before. Therefore, the health system and institutions are forced to adapt to the changes in economy, society, market, and regulation. As the main provider of health services in China, public hospitals are facing great pressures related to the increasing demand for the medical services. Therefore, policy-makers need to increase the efficiency and effectiveness of the use of the scarce medical resources. 

China has been implementing the third round of reforms to the medical system since 2009. Differently from the previous two rounds of health care reform, this round does not stress the social benefits, yet seeks for balance between the social benefits and efficiency of the public hospitals. Since 2009, the Chinese government gradually carried out a series of reforms in public hospitals to improve their performance, including the establishment of the basic medical security system, national essential medicine system, improving the primary medical and health service system, promoting the gradual equalization of basic public health services, and the pilot reform of public hospitals.

To increase the bargaining power of the demand side, China established the basic medical insurance program with the widest coverage in the world in 2018. The program allows exploiting the scale effect as it is the largest healthcare service buyer and is able to share the underlying risk among the insured. To control the rapid surge in medical service prices, the drug markup policy in public hospitals had been gradually phased out in some pilot public hospitals since 2009 and eventually abolished nationwide in 2017. The resulting revenue gap is to be covered through the increases in prices of health service, financial subsidies and health insurance. The regulation concerning the price of health service was relaxed. Basic health services were priced according to guidelines set by the regional government while others were priced at the discretion of the public hospitals. To increase the competition in the public hospital sector in China, Chinese government encouraged for-profit hospitals to compete with public hospitals in 2010. Public hospitals were directed towards maintaining public welfare and non-profit activities in 2009. In addition, public hospitals generate revenue by providing health services and public services to patients instead of getting financial subsidy from government directly. The role of government changed from supply side to demand side. The technical efficiency assessment of public hospitals, implying maximum output for minimum input, is an important facet of healthcare sector performance and can provide evidence on the effects of the abovementioned reforms in China. 

Data envelopment analysis (DEA) is a versatile tool able to assess the relative efficiency. As such, it has been widely adopted in studies on the health industry. Relying on non-parametric estimation, DEA is suitable for economic analysis when parametric methods face certain obstacles. For example, DEA assumes some basic axioms of production theory without assuming particular functional form of the production function [1], which is required by Stochastic Frontier Analysis (SFA) as a parametric method. Even so, DEA still has drawbacks. The conventional DEA only estimates efficiency of decision making units (e.g., public hospitals), but cannot identify the impact of environmental factors and statistical noise on efficiency, which can be taken into account by the SFA. Therefore, the three-stage DEA approach is employed in the paper. The latter approach is able to overcome the aforementioned shortcomings. DEA is used to estimate the efficiency of public hospitals producing multiple outputs with multiple inputs. For the reasonable assessment, it is crucial to select appropriate inputs and outputs. Bin et al. [2] divided input variables into three categories: physical, human and financial resources. Some studies did not incorporate financial resources into input variables [3,4], whereas some studies attached great importance to human resources and distinguished between different types of personnel [5]. In addition, the number of beds is often used as a proxy of physical resources [2,3,4].

The objective of the paper is to evaluate the efficiency of public hospitals and take into consideration the influence of environmental factors and statistical noise for the case of China. The paper adds to the existing literature in the two aspects, namely method applied and the output variables used. First, to avoid over- or underestimating the efficiency of public hospitals, we employ the three-stage DEA to assess the efficiency of public hospitals. We take the regions as the decision making units, which allows capturing the effects of public hospital reform across different regions during the period covered. Second, the outputs for efficiency assessment include positive externalities reflecting public welfare gains due to the public hospitals. This represents the objectives of Chinese government and is seldom reflected in the previous literature. This study employs the three-stage DEA to estimate the efficiency of the regional public hospitals covering 31 regions in China from 2011 to 2018. The empirical results provide policy implications for decision-makers.

In the next section of the paper, we review research on the assessed efficiency of healthcare providers, especially public hospitals in China. Then, the methodological approach is introduced in Section 3, followed by the data sources and indicators presented in Section 4. The empirical results are given in Section 5. We conclude with a discussion and policy recommendations in Section 6.

## 2. Literature review

### 2.1. DEA Literature

It is crucial for accurate evaluation to select the appropriate method. In efficiency analysis, relative performance evaluation is a basis to identify the performance gaps. The technical efficiency refers to how well a public hospital uses inputs to produce outputs. The more outputs are produced with a given amount of inputs, the higher the efficiency. Alternatively, less inputs are used to produce a given amount of outputs, the higher the efficiency. Two different approaches are often employed to evaluate the institution efficiency: SFA and DEA. Both of the methodologies infer efficiency from a residual. The difference between them is that DEA assumes that departures from efficient frontier reflect inefficiency while SFA assumes that the residual reflects inefficiency and random factors. However, SFA requires assuming particular functional form of the production function, though it can analyze the impact of environmental factors and statistical noise on efficiency. Wrongly specified functional form may produce distorted results. Accordingly, DEA is often used as a methodology to assess the efficiency of public hospitals.

The different methods based on DEA have been adopted to evaluate the efficiency of institutions and health system in general. The conventional DEA was employed to gauge the technical efficiency of public hospitals in India [6], Jordan [7,8], Portugal [9], Austria [10], and China [2,11] with input or output orientation. The literature on Chinese public hospital efficiency focuses on the third round of medical reform in China since 2009. Bin et al. [2] adopted DEA to assess efficiency of 19 tertiary general public hospitals in Tianjin and found that the majority of the observations were technical and scale efficient and that health resource constraints and resource waste coexist. Xu et al. [11] used three methods, including DEA, to measure the efficiency of 50 tertiary public hospitals in Beijing. 

Another strand of literature focuses on efficiency assessment of health institutions by means of the two-stage DEA. Such an approach allows analyzing the impact of efficiency factors [3,12,13,14,15,16,17], such as reforms of health insurance [3], payment system of medical insurance [12], finance management [17]. In addition, Cho et al. [14] also employed the two-stage approach to evaluate the impact of health information technology adoption and hospital-physician integration on hospital efficiency. Afonso and St. Aubyn [15] used DEA to assess health efficiency of 30 OECD countries and regressed efficiency scores on environmental variables. Hu et al. [3] used two-stage DEA to investigate the regional hospital efficiency before and after health insurance reform of New Rural Cooperative Medical System and the impact of the reform on the efficiency of hospitals in China. Jing et al. [4] employed DEA to evaluate the technical efficiency of public and private hospitals in Beijing and analyzed the factors of technical efficiency.

With few exceptions, most of the previous studies employed the conventional DEA or two-stage DEA to examine the efficiency and its factors of the healthcare systems. DEA and SFA have their own disadvantages, which are easy to overcome in the three-stage DEA approach. The three-stage DEA, proposed by Fried et al. [18], takes the environmental effects and statistical noise into account throughout efficiency assessment. This can provide more accurate efficiency scores and allow eliminating the impact of statistical noise and putting decision-making units (DMUs) under the same external environment. 

### 2.2. Output Variables

Another important issue in efficiency analysis is to determine the input and output variables that characterize the production process in the best possible way. Inputs should incorporate all the necessary resources, and outputs need to describe the managerial objectives of the DMU [19]. The research on hospital performance have little difference in the choice of inputs, mainly including physical, human and financial resources [2]. As regards the choice of outputs, there can be more variety besides the outpatients and inpatients being treated as outputs. Such outputs as patient mortality can enter the model as undesirable outputs [3], successful rescue of critical patients—as a measure of the outputs quality [20]. The selected DEA studies and outputs used are shown in Table 1. Most of the existing literature assumes that the outputs of public hospitals were the same as that of other types of hospitals (i.e., the same outputs are used).

According to China’s strategy towards the public hospitals, they not only undertake the task of providing basic medical services, but also embark on the tasks related to public welfare (e.g., infectious disease prevention, health education, disaster relief). The latter dimension is often neglected in the previous studies. Mead Over and Naoko Watanabe [24] divided the functions of public hospital into social functions and private functions. Social functions refer to the outputs that serve objectives beyond those of the individual patient, including public goods and goods with positive externalities. The examples of such services include dissemination and research, treatment of communicable diseases and a healthcare safety net. Private function is the health services providing benefits for an individual patient. If the choice of the outputs does not conform to the managerial objectives of public hospitals, the results of efficiency analysis will obviously be biased. Therefore, the outputs corresponding to social functions must be incorporated into the outputs. 

## 3. Methods

Fried et al. [18] argued that inefficiency of decision-making units is attributable to managerial inefficiency, environmental factors and statistical noise. These factors are necessary to separate from each other for accurate efficiency assessment. Therefore, the three-stage DEA method was proposed.

### 3.1. Stage 1: Input-Oriented DEA

In the first stage, the conventional DEA is implemented using the original input and output data. We apply the constant returns to scale input-oriented DEA that corresponds to the cost minimization problem. The efficiency scores for each DMU can be obtained by solving the following linear programming problem:

(1) $$\begin{array}{l} \min\theta -\varepsilon \left(1S^{-}+1S^{-}\right)\\ s.t.\\ \sum_{j=1}^{n}X_{j}\lambda_{j}+S^{-}=\theta X_{0}\\ \sum_{j=1}^{n}Y_{j}\lambda_{j}-S^{+}=Y_{0}\\ \lambda_{j}\geq 0,S^{-},S^{+}\geq 0 \end{array}$$

where θ represents the efficiency score bounded at unity from below; *j* is the index of DMUs *,* j=1,2,⋯,n and ${X\;}_{j}$ and ${Y\;}_{j}$ represent input vector and output vector of ${\mathrm{DMU}}_{j}$ respectively; $S^{+}$ and $S^{-}$ denote non-radial slack variables for outputs and inputs respectively; $\lambda_{j}$ is a weight of ${\mathrm{DMU}\;}_{j}$ (intensity variable). Thus, the total slack comprises the radial part captured by θ and the non-radial part captured by S.

### 3.2. Stage 2: Effects of the Environmental Variables 

In the second stage, the slacks of inputs obtained from (1) are used as the explained variables that are regressed on environmental variables. Subsequently, the original input data are adjusted according to the regression results. If the input slacks are regressed separately, the different impact of environmental variables on each input can be assessed at the cost of degrees of freedom [25]. The SFA formulation is applied according to Fried et al. [18] as follows:

(2) $$S_{ij}=f(Z_{j};\beta_{i})+\nu_{ij}+\mu_{ij};j=1,2,\cdots ,n;i=1,2,\cdots ,I,$$

where $S_{ij}$ represents the slack of input *i* of the *j*-th DMU; ${Z\;}_{j}$ denotes the environmental variables for DMU j; $f(Z_{i};\beta_{n})$ represents function (impact) of environmental variables affecting input slack $S_{ij}$, the linear regression is considered so that $f(Z_{j};\beta_{i})=\beta_{i}\cdot {Z\;}_{j}$; $\nu_{ni}+\mu_{ni}$ represents composite error term, with $\nu_{ni}$ and $\mu_{ni}$ representing the statistical noise and managerial inefficiency respectively. Assuming $\nu \sim N(0,\sigma_{v}{}^{2})$ and $\mu \sim N^{+}(0,\sigma_{\mu }{}^{2})$, let $\gamma =\sigma_{\mu i}^{2}/(\sigma_{\mu i}^{2}+\sigma_{\nu i}^{2})$. The closer the value of γ is to 1, the more managerial inefficiency dominates the composite error of the model.

Fried et al. [18] decomposed mixed error term according to Jondrow et al. [26]. However, Jondrow et al. [26] adopted the composite error of the production function ε=ν−μ, whereas the consideration of the input slacks requires the form of the cost function with the composite error ε=ν+μ. The random error is obtained according to Luo [27] as:

(3) $$E\left(\mu \left|\varepsilon \right.\right)=\sigma_{*}\left[\frac{\phi (\lambda \frac{\varepsilon }{\sigma })}{\Phi (\frac{\lambda \varepsilon }{\sigma })}+\frac{\lambda \varepsilon }{\sigma }\right]$$

where $\sigma_{*}=\frac{\sigma_{\mu }\sigma_{\nu }}{\sigma }$, $\sigma =\sqrt{\sigma_{\mu }{}^{2}+\sigma_{\nu }{}^{2}}$ and $\lambda =\sigma_{\mu }/\sigma_{\nu }$; $\phi \left(\cdot \right)$ and $\Phi \left(\cdot \right)$ are the density function and distribution function for the standard normal variables respectively. 

The adjusted input value is obtained through

(4) $$X_{ij}^{A}=X_{ij}+[\max(f(Z_{j};{\overset{\wedge }{\beta }}_{i}))-f(Z_{j};{\overset{\wedge }{\beta }}_{i})]+[\max(\nu_{ij})-\nu_{ij}],j=1,2,\cdots ,n;i=1,2,\cdots ,I$$

where $X_{ij}$ and $X_{ij}^{A}$ represents the original input quantity and the adjusted input quantity respectively; $\left[\max(f(Z_{i};{\overset{\wedge }{\beta }}_{n}))-f(Z_{i};{\overset{\wedge }{\beta }}_{n})\right]$ adjusts the DMU to external environment; $\left[\max(\nu_{ni})-\nu_{ni}\right]$ adjusts the DMU with regards to the statistical noise. The adjustments place the DMUs (regions) in the most beneficial situation with regards to the operation environment and statistical noise. 

### 3.3. Stage 3: Recalculation of the Efficiency Scores

In the third stage, we replace the original input quantities with the adjusted input quantities from (3), and repeat the first stage analysis by applying DEA in (1) to the adjusted data. Then, the efficiency scores from the third stage are compared to those from the first stage. 

## 4. Data 

This paper uses the following inputs:

(1) The number of licensed (assistant) doctors.
(2) The number of other technical staff, including registered nurses, pharmacists, and other technicians.
(3) The number of managers.
(4) The number of beds.

With regard to the selection of outputs, we add an output variable reflecting the output of public hospitals’ social functions besides outpatients and inpatients services. Still, there is no consensus on the measurement of social functions (output). Jin and Song [28] used incidence rate of infectious diseases as a measure of social function (output) of public health sector. Following Jin and Song [28], we use the number of infectious patients treated in public hospitals as a measure of social function (output). The infectious diseases refer to class A and B notifiable infectious diseases in China covering 27 species, including plague, cholera, viral hepatitis, syphilis. Thus, the outputs in our study include:

(1) The number of outpatient visits.
(2) The number of inpatients visits.
(3) The number of infectious patients treated in public hospitals.

Generally, the number of DMUs is required to be twice the sum of the numbers of input variables and output variables [19]. This condition is met in this study (31 > 2 × (4 + 3)). The descriptive statistics for inputs/outputs are given in Table 2.

In the DEA model, input variables and output variables should satisfy the monotonicity property, i.e., the outputs should not decrease with increase in input quantities. The Pearson correlation coefficient is employed to examine this issue (Table 3). The correlation coefficients among inputs and output are positive and range from 0.665 to 0.955. This indicates that the choice of the input and output indicators conforms to the theoretical requirements.

Hospital performances is not only influenced by the controllable factors, such as inputs and outputs, but also by environmental variables beyond its control. Hu et al. [3] categorized the environmental variables influencing performance of public hospitals into market conditions, ownership, and regulation of government. Based on the aforementioned approach, we consider the following environmental variables for China’s public hospitals:

(1) The price of medical services. The high levels of medical service price have been a common concern in China. Indeed, high prices of the health services induce economic burden resulting in reduction in demand for medical services. The expenditure per outpatient visit and expenditure per inpatient visit are used to measure the price of outpatient services and the price of inpatient service respectively.
(2) Insurance penetration. Due to the differences in medical insurance plans, the ratio of the covered by the basic medical insurance to population in a region is adopted to reflect the reform of medical insurance. The insurance penetration can affect both supply and demand side of medical services. As a professional third buyer, the insurance can force public hospitals to improve efficiency and quality of medical services, because insurance can adjust the patients’ demand for some hospital’ medical services via the reimbursement policies. Increasing insurance penetration allows sharing economic risks among the insured, so that patients access the medical services in a timely manner. Thus, reasonable medical insurance is expected to be positively related to the efficiency of public hospitals.
(3) Public subsidies. Public subsidies represent government’s responsibility for medical service. The subsidy is measured by the ratio of government subsidies to the total public hospitals’ income. Public subsidies are expected to improve efficiency through hiring more skilled staff and purchasing advanced equipment. Still, a moral hazard problem may arise as public hospitals can survive even if they reduce their efforts to produce high-quality medical services.
(4) Competition. Chinese government expects public hospitals to improve their performance through competition with for-profit hospitals. The ratio of the number of for-profit hospitals to total hospitals in a province is employed to measure the intensity of competition. The for-profit hospitals are expected to force public hospitals to get more revenue by improvement in quality and reduction in cost, which lead improvement in efficiency.
(5) Quality of health services. The ratio of tertiary public hospitals to total hospitals in a region is employed to measure the quality of health services in the market. The higher the ratio, the better the quality of health services is expected. The tertiary hospitals can combinate the advanced medical equipment and skilled staff to provide more healthcare services. So, quality of health services is also expected to be positively correlated with hospital efficiency.
(6) Demand for medical services. Children and elderly are likely to exhibit higher demand for health services. Thus, the ratio of population aged 0–14 and above 65 years to the total populations in a region is used to measure the demand for health service. In general, the increase in demand for medical service may lead to more skilled doctors, resulting in improvement in efficiency of public hospitals.

The environmental variables are described in Table 4 and Table 5.

Since 2009, China has been implementing the third round of the health system reform. Once the reform policies are effective, a certain lag period is required to manifest their effects. Accordingly, 2011 is selected as the starting year for the analysis. The most recent data can be obtained for 2018. Accordingly, the period of 2011–2018 is chosen for the analysis. The province-level data describing performance of the public hospitals are obtained from the National Bureau of Statistics of China and Statistical Information Center of the National Health Commission. Due to absence of relevant data of Tibet in 2012, this paper applies interpolation in this case.

## 5. Results

The efficiency scores based on (1) for 31 regions during 2011–2018 are given in Table 6. The efficiency scores in the first and third stages are estimated separately for each year. The results are provided in Table 6 and Table 9. Note that the second stage assumes independent distribution of the error terms and utilizes the pooled data.

In general, the performance of regional public hospitals in China slightly decreased during the period of 2011–2018 as the mean efficiency score declined from 0.926 in 2011 down to 0.915 in 2018. The inverse U-shaped trend peaking in 2015 is observed for the mean efficiency scores. Indeed, it was in 2015 that China entered the “new normal stage” of economy when the growth rate of government investment in the health system began to decline. The numbers of efficient regions range from 13 to 17. This is related to relatively high number of the input and output variables. Eleven regions are fully efficient during 2011–2018. These regions include Shanghai, Zhejiang, Shandong, Hunan, Guangdong, Guangxi, Yunnan, Tibet, Gansu, Qinghai, and Xinjiang. It is found that the efficiency score of regional public hospitals in Shanxi is the lowest among the regions (0.653). In the first stage, the efficiency of public hospitals may be biased due to differences in the operation environment and statistical noise. We proceed to the SFA-based analysis to isolate the managerial inefficiency (i.e., performance in transforming inputs into outputs) from effects of the environmental variables and statistical noise.

Table 7 presents the average slacks (including both radial and non-radial part) for the four inputs. These values indicate the possible contraction in the input quantities for a given technology and output levels. The annual results are presented in Appendix A.

Table 8 shows the SFA results for the second stage analysis. The likelihood ratio test indicates that each regression is significant at 1% significance level, indicating that it is reasonable to apply the SFA model. In addition, most of the estimated coefficients for environmental variables pass the significance test at acceptable level of significance. Therefore, the environmental variables exert significant effects on the slacks and, hence, technical efficiency.

The positive regression coefficients for environmental variables imply that an increase in the environmental variable will result in an increase in the slack variable under evaluation. Thus, an increase in the environmental variable will further increase the input redundancy to and reduce the efficiency of public hospitals. On the contrary, a negative regression coefficient of environmental variables to some slack variable implies that the increase in environmental variable will result in a decrease in the slack variable which will improve the performance of public hospitals.

In all the models, regression coefficients associated with the average medical expenses of outpatients are significantly positive at the 1% significance level, indicating positive correlation between average medical expenses of outpatients and input slacks. An increase in the price of outpatient services will lead to the increase of the input redundancy in licensed doctors, registered nurse, other technical staff, and beds, which had negative effects on the efficiency of public hospitals. The possible reason is that the high price of outpatient service hinders the patients’ access to outpatient service, resulting in idle resources and reducing the efficiency of public hospitals.

The regression coefficients for the average medical expenses of inpatients are negative and significant (except for licensed doctors). The increase in the price of inpatient services results in the decrease of the input redundancy in registered nurses, other technical staff, and beds. Such changes lead to improved efficiency of public hospitals. China’s government reduced the prices of outpatient and inpatient services simultaneously during the latest round of the reform. However, the difference between the prices of outpatient and inpatient service is so small that patients with common diseases and chronic diseases tend to replace outpatient services with inpatient service, which results in congestion of inpatient services which undermines the efficiency of public hospitals.

The regression coefficient for Insura is negative and statistically significant in the input slack of beds regression, whereas the corresponding regression coefficients in the regressions for other input slack variables are positive. Thus, Insura exerts two opposite effects: the increase in the ratio of the covered by the basic medical insurance to the total population is accompanied by a decrease in the input redundancy of beds (which increases efficiency of public hospitals), and increase in the input redundancy of licensed doctors, registered nurses, other technical staff which reduced the technical efficiency. The aim of improving the efficiency of public hospitals by increasing the penetration of the health insurance may not be achieved in case the existing insurance systems are not redesigned.

Public subsidies show significant negative impact on the slacks of licensed doctors and number of beds, whereas significantly positive effects are observed for other inputs. It may be explained by the fact the public subsidies are likely to have dual effects towards opposite directions. The Chinese government subsidizes public hospitals on a per-person basis, rather than financing healthcare services, which may be the cause of the problem. Thus, the Chinese government needs to reform the subsidy schemes to induce efficiency gains in the public hospitals.

Competition show no significant effect on technical staff (even though a negative value is observed), whereas the other coefficients are significantly positive suggesting that the increase in the intensity of competition will be accompanied by an increase in the input redundancy of licensed doctors, registered nurses, and beds. Obviously, this pattern indicates that increasing competition tends to decrease the efficiency of public hospitals. China’s government expects to public hospitals to improve their efficiency through competition with for-profit hospitals. However, due to apparent monopoly of the public hospitals, the for-profit hospitals do not compete directly with public ones, rather embark on the monopolistic competition by providing services that are not available at the public hospitals. This situation does not provide the public hospitals the incentives to improve their performance.

The quality of health services (as represented by the share of the tertiary level hospitals) affects negatively all the input slack variables (significant at the 1% level). The increase in the quality of health services reduces the input redundancy thereby improving the performance of public hospitals. The higher ratio of tertiary public hospitals to total hospitals implies that more skilled staff and advanced equipment are available in a certain province which induces efficiency gains.

Similar to the case of the quality of medical services, the regression coefficients on demand for medical services are significantly negative (at the 1% level) against all the inputs. Accordingly, an increase in demand for medical services is to be associated with a reduced input redundancy and improved performance of the public hospitals. A region with the higher ratio of population aged 0−14 and above 65 years to the total populations exhibits higher demand for medical services and, likely, a higher level of efficiency.

In the second stage, we isolated statistical noise according to (2) (inefficiency term in the SFA model remained ignored), and obtained the adjusted input quantities that take into account the impact of environmental variables and statistical noise as per (3). Then, the third stage embarked on calculation of the efficiency scores by exploiting (1) and the adjusted input quantities along with the initial output quantities. The adjusted efficiency scores are presented in Table 9.

Comparison of data in Table 6 and Table 9 suggests that the average of efficiency of the for 31 regions increased up to 0.981 from 0.927 due to adjustment in accordance with the environmental variables and statistical noise. This implies that efficiency of public hospitals was underestimated in the first stage, i.e., the operational environment affects the input quantities in a negative manner: input quantities are increased to take the environment-implied constraints into account which renders lower levels of efficiency. Even, so, the efficiency change trend persists as the mean efficiency scores reported in Table 9 follow the inverse U-shaped trend. The average of number of efficient DMUs also increases from 15.25 to 18.75 after input variables are adjusted via the three-stage procedure.

As for individual regions, 11 regions remained fully efficient over the period covered after implementing the three-stage procedure. The regions with fully efficient public hospital operation (after adjustment for environmental conditions) include Tianjin, Shanghai, Zhejiang, Shandong, Hunan, Guangdong, Yunnan, Tibet, Gansu, Ningxia, and Xinjiang. Compared with the regions on the efficient frontier in the first stage, Guangxi and Qinghai were replaced by Tianjin and Ningxia. It can be also found that the efficiency values of 17 regional public hospitals are underestimated, including those in Beijing, Tianjin, Hebei, Shanxi, Inner Mongolia, Liaoning, Jilin, Heilongjiang, Jiangsu, Anhui, Fujian, Hainan, Chongqing, Sichuan, Guizhou, Shaanxi, and Ningxia in case the environmental variables are not accounted for. Public hospitals in Shanxi have the poorest performance (0.899) after accounting for environmental context, yet this province experienced the highest increase in the efficiency score (the change of 0.246). In addition, the efficiency scores of 5 regions are overestimated in case the environmental factors are not taken into account: Jiangxi, Henan, Hubei, Guangxi, and Qinghai. The efficiency score for Jiangxi declined to the highest extent (from 0.991 to 0.983) during the three-stage procedure.

## 6. Conclusions

The three-stage DEA was employed to assess the efficiency of public hospital performance across 31 regions in China during 2011–2018. The inputs included the numbers of licensed doctors, registered nurses, other technical staff and beds. The output variables included the numbers of outpatients, inpatients and infectious patients. Note that the output variables used in this study reflect both the private and social functions. Indeed, the latter functions were often ignored in the previous literature.

The results showed that the efficiency across and ranking of the regions is altered due to the introduction of the environmental variables describing the operation context of the public hospitals across the regions. Therefore, it is necessary to eliminate the effects of environmental factors and statistic noise when assessing the performance of China’s public hospitals in order to obtain robust results. In general, the efficiency of public hospital operation was underestimated. The mean efficiency score increased from 0.927 to 0.981 after accounting for the impact of environmental variables and statistical noise. The trend of the efficiency scores followed an inverted U-shape with an upturn during 2011–2015 and a decline thereafter.

Most of environmental variables adopted in this analysis had significant effects on the performance of public hospitals. The government should reasonably set the price of medical service and appropriately expand the price gap between outpatient service and inpatient service. For the patients’ accessibility to medical services, the Chinese government attempted to keep the prices of outpatient service and inpatient service unchanged or to even reduce the prices by price regulation, which will damage the performance of public hospitals according to the viewpoint of this paper. The government should decrease the price of outpatient service to ensure the patients’ accessibility, and increase the price of inpatient service to prevent outpatients’ abusing inpatient services due to the difference in reimbursement plan.

The results indicate that the Chinese government should change the subsidy basis in order to avoid different treatment of the provinces. As an important means of regulation, public subsidies should act as an impetus to induce efficiency gains in the public hospitals. Currently, however, the moral hazard occurs and renders negative effects of the subsidies. Accordingly, the Chinese government is suggested to subsidize public hospitals on the basis of the healthcare services provided rather than on a per person basis. In addition, higher subsidies should be allocated to the social functions of the public hospitals. This will allow producing safety nets though prevention and treatment of infectious diseases that are becoming more and more important.

The insurance schemes should also be redesigned. The insurance can influence the patients’ demand for medical services through reimbursement plan. A reasonable reimbursement plan can guide patients to visit to hospital dependent on their condition. Patients with mild diseases should be encouraged to visit the primary hospitals for outpatient services while ones with sever diseases visit to the second-level hospitals and tertiary hospitals for inpatient services. In addition, the insurance can force public hospitals to improve their performance, as insurance can adjust patients’ demand for certain healthcare services through reimbursement schedules. So far, insurance needs to be further improved as the environment of the public hospital operation is distorted by the presence of the currently existing insurance systems.

This paper focused on the efficiency patterns. Further research could attempt to identify the productivity change and its drivers in order to gain insights into the dynamics of the public hospital performance in China. The sources of productivity growth could also be identified. Furthermore, different sets of the input and output variables could be tested.

## Figures and Tables

**Table 1:** Selected DEA studies.

Reference	Context	Outputs
Li et al. [21]	Public hospitals in the Philippines	Total patients; Laboratory services; Net Death Rate
Hu et al. [3]	Chinese regional hospitals	The total number of outpatient and emergency room visits; The total number of inpatient days; Patient mortality (undesired outputs).
Ajlouni et al. [7]	Jordanian public hospitals	The annual number of patient days; Number of minor surgical operations per year; Number of major surgical operations per year.
Barnum et al. [22]	Community hospitals in the Units States	Annual Inpatients; Annual Outpatients.
Giénez et al. [23]	Colombian public hospitals	sum of the weighted services provided by hospitals’ relative value unit.
Paul and Steffen [17]	The primary care facilities in rural Burkina Faso	General consultation and nursing care; Deliveries; Immunization; Special services.
Sultan and Crispim [8]	Jordanian public hospitals	Inpatient days; Outpatient services; Ambulance and emergency services.
Bin et al. [2]	Public hospitals in China	Number of outpatient and emergency visits; Number of discharged inpatients; Total revenue.
Jing et al. [4]	Public hospitals in China	Outpatient and emergency visits; Inpatient discharges; Revenue.
Xue [20]	China’s health care system	Outpatient visits; Discharged inpatients; Surgical operations; Successful rescue of critical patients.

**Table 2:** Descriptive statistics for inputs and outputs (2011–2018).

Category	Variable	Mean	S.D.	Min	Max
**Inputs**	Licensed doctors	53,060	34,186	2341	166,567
	Registered nurses	92,026	59,535	1951	279,184
	Other technical staff	9661	5593.021	367	23,898
	Beds	164,182	106,077	5385	460,703
**Outputs**	Infectious patients	100,300	68,820	7061	387,240
	Outpatient	84,499,415	67,081,486	2,901,304	339,038,474
	Inpatients	4,275,422	2,775,257	101,510	12,005,845

**Table 3:** Pearson correlation coefficients for input and output indicators.

Correlates	Licensed Doctors	Registered Nurse	Other Technical Staff	Beds
**Outpatient**	0.692	0.846	0.772	0.757
**Inpatient**	0.767	0.955	0.861	0.946
**Infectious patients**	0.689	0.718	0.665	0.704

**Table 4:** Description of the environmental variables.

Notation	Unit	Definition
Oprice	Yuan per visit	Average medical expenses of outpatients
Iprice	Yuan per visit	Average medical expenses of inpatients
Insura	%	ratio of the insured of basic medical insurance to population
Subsid	%	ratio of government subsidy to the public hospital income
Compet	%	ratio of the number of for-profit hospitals to total hospitals
Qualit	%	ratio of tertiary public hospitals to total hospitals
Demand	%	ratio of population aged 0–14 and above 65 years to populations

**Table 5:** Descriptive statistics for environmental variables.

Variable	Mean	S.D.	Min	Max
Oprice	218.18	62.42	75.4	530.9
Iprice	8574.63	3241.69	3906.3	22645.8
Insura	0.4677	0.2538	0.1286	1.084
Subsid	0.3267	0.0947	0.2009	0.7155
Compet	0.4780	0.1504	0.0485	0.7846
Qualit	0.0774	0.0312	0.0189	0.1574
Demand	0.264	0.035	0.162	0.336

Note: The basic medical insurance includes medical insurance for urban and rural residents, medical insurance for urban employees, and free medical services for retirees. In case the categories of insurance overlap for some residents, the insurance penetration ratio can exceed 1. For example, an employee retires in the middle of the year, which is recorded twice that year. However, due to the small proportion, it does not affect the measurement of the insured ratio.

**Table 6:** The efficiency scores of regions in the first stage (2011–2018.).

Province	2011	2012	2013	2014	2015	2016	2017	2018	Mean
Beijing	0.91	0.92	0.963	0.933	0.961	0.997	0.846	0.915	0.931
Tianjin	0.969	0.898	0.887	0.882	0.861	0.875	0.839	0.849	0.883
Hebei	1	1	1	1	0.989	1	0.974	0.923	0.986
Shanxi	0.612	0.664	0.669	0.681	0.646	0.661	0.621	0.67	0.653
Inner Mongolia	0.765	0.752	0.739	0.729	0.707	0.727	0.728	0.792	0.742
Liaoning	0.74	0.764	0.803	0.825	0.852	0.831	0.81	0.748	0.797
Jilin	0.773	0.799	0.801	0.814	0.798	0.739	0.774	0.751	0.781
Heilongjiang	0.731	0.741	0.778	0.79	0.827	0.842	1	0.804	0.814
Shanghai	1	1	1	1	1	1	1	1	1
Jiangsu	0.851	0.82	0.85	0.866	0.872	0.885	0.841	0.827	0.852
Zhejiang	1	1	1	1	1	1	1	1	1
Anhui	0.9	0.86	0.881	0.898	0.929	0.903	0.933	0.884	0.899
Fujian	1	1	1	1	1	1	1	0.971	0.996
Jiangxi	0.982	0.962	0.99	1	1	1	1	0.997	0.991
Shandong	1	1	1	1	1	1	1	1	1
Henan	1	1	1	1	1	0.988	0.983	1	0.996
Hubei	1	0.993	1	1	1	1	1	1	0.999
Hunan	1	1	1	1	1	1	1	1	1
Guangdong	1	1	1	1	1	1	1	1	1
Guangxi	1	1	1	1	1	1	1	1	1
Hainan	0.96	0.919	0.988	0.979	0.968	0.975	1	0.998	0.973
Chongqing	0.92	0.913	0.885	0.883	0.895	0.787	0.857	0.765	0.863
Sichuan	0.96	0.95	0.943	0.918	0.967	1	0.584	0.878	0.900
Guizhou	0.864	0.817	0.868	0.906	0.898	0.818	0.889	0.829	0.861
Yunnan	1	1	1	1	1	1	1	1	1
Tibet	1	1	1	1	1	1	1	1	1
Shaanxi	0.816	0.838	0.846	0.857	0.885	0.834	1	0.841	0.865
Gansu	1	1	1	1	1	1	1	1	1
Qinghai	1	1	1	1	1	1	1	1	1
Ningxia	0.96	1	0.969	0.943	0.996	0.922	0.894	0.93	0.952
Xinjiang	1	1	1	1	1	1	1	1	1
Mean	0.926	0.923	0.931	0.932	0.937	0.929	0.922	0.915	0.927
Efficient DMUs	15	15	15	16	15	16	17	13	15.25

**Table 7:** Mean values of the four slack variables for 2011–2018.

Province	Licensed Doctors	Registered Nurse	Other Technical Staff	Beds
Beijing	18,950	22,145	4996	7204
Tianjin	6802	4875	3437	6608
Hebei	11,108	2285	357	9555
Shanxi	23,369	28,125	3576	47,538
Inner Mongolia	10,718	14,457	2387	25,941
Liaoning	15,665	21,951	3672	49,541
Jilin	11,441	12,293	4350	25,662
Heilongjiang	11,507	14,409	4596	32,082
Shanghai	0	0	0	0
Jiangsu	15,741	26,406	2593	62,834
Zhejiang	0	0	0	0
Anhui	6442	11,369	960	19,550
Fujian	344	1444	25	531
Jiangxi	669	2788	46	881
Shandong	0	0	0	0
Henan	437	780	474	9921
Hubei	102	83	29	248
Hunan	0	0	0	0
Guangdong	0	0	0	0
Guangxi	0	0	0	0
Hainan	951	4136	613	674
Chongqing	4552	9177	2606	29,969
Sichuan	9965	19,819	4688	63,627
Guizhou	5001	9375	1946	30,932
Yunnan	0	0	0	0
Tibet	0	0	0	0
Shaanxi	6302	14,591	5738	24,033
Gansu	0	0	0	0
Qinghai	0	0	0	0
Ningxia	566	1641	121	1640
Xinjiang	0	0	0	0

**Table 8:** The SFA results for the second-stage analysis.

Environmental Variables	Input Slack Variables			
	Licensed Doctors	Registered Nurses	Other Technical Staff	Beds
Constant	17,123.43 *** (713.82)	21,039.96 *** (1453.02)	5762.96 *** (345.2)	21,653.04 *** (1.06)
Oprice	38.08 *** (14.32)	63.35 *** (18.67)	18.38 *** (4.6)	88.86 ** (43.13)
Iprice	−0.37 (0.29)	−1.2 *** (0.38)	−0.26 *** (0.07)	−1.72 * (1.02)
Insura	2155.1 * (1260.77)	3207.5 * (1721.54)	565.8 (390.04)	−4801.3 *** (1.01)
Subsid	−1723.9 *** (308.9)	4163.1 *** (591.78)	968.3 *** (283.61)	−21,164.7 *** (1.01)
Compet	5609.2*** (2.07)	11,418.7*** (812.85)	−559.5 (1013.59)	48,806.9 *** (1.04)
Qualit	−72,477.4 *** (49.34)	−58,172.4 *** (122.63)	−14,628.4 *** (649.94)	−102,167.1 *** (1)
Demand	−79,566.6 *** (245.47)	−108,809.2 *** (408.48)	−26,107.1 *** (1098.74)	−141,534.5 ***(1)
Sigma-squared	74,340,902 *** (1)	149,417,560 *** (1)	5,798,688.6 *** (2.12)	890,435,570 *** (1)
γ	0.79 *** (0.02)	0.79 *** (0.02)	0.83 *** (0.01)	0.77 *** (0.02)
Log likelihood	−2454.19	−2533.56	−2101.38	−2761.6
One-sided LR Test	132.22 ***	143.60 ***	180.09 ***	133.2 ***

Note: *, **, *** denote significance at 10%, 5%, and 1% statistical levels respectively; Figures in parentheses are standard errors.

**Table 9:** The efficiency scores of 31 regional public hospitals adjusted for operation environment and statistical noise (2011–2018).

Province	2011	2012	2013	2014	2015	2016	2017	2018	Mean
Beijing	1	1	1	1	1	1	0.977	1	0.997
Tianjin	1	1	1	1	1	1	1	1	1
Hebei	1	1	1	1	1	1	1	0.974	0.997
Shanxi	0.899	0.929	0.9	0.912	0.891	0.901	0.861	0.898	0.899
Inner Mongolia	0.986	0.961	0.935	0.934	0.931	0.939	0.907	0.962	0.944
Liaoning	0.879	0.897	0.925	0.941	0.948	0.943	0.926	0.879	0.917
Jilin	0.957	0.977	0.983	0.98	0.975	0.95	0.955	0.945	0.965
Heilongjiang	0.909	0.907	0.931	0.941	0.955	0.96	1	0.947	0.944
Shanghai	1	1	1	1	1	1	1	1	1
Jiangsu	0.947	0.914	0.92	0.936	0.935	0.935	0.905	0.897	0.924
Zhejiang	1	1	1	1	1	1	1	1	1
Anhui	0.967	0.969	0.971	0.986	0.976	0.974	0.968	0.96	0.971
Fujian	1	1	1	1	1	1	1	0.998	1.000
Jiangxi	0.964	0.965	0.975	1	1	1	0.972	0.989	0.983
Shandong	1	1	1	1	1	1	1	1	1
Henan	1	1	1	1	1	0.984	0.985	1	0.996
Hubei	0.998	0.994	0.994	1	1	1	1	1	0.998
Hunan	1	1	1	1	1	1	1	1	1
Guangdong	1	1	1	1	1	1	1	1	1
Guangxi	1	1	1	1	1	1	0.991	1	0.999
Hainan	0.986	0.998	1	1	0.989	0.989	1	1	0.995
Chongqing	0.991	0.99	1	1	1	0.964	1	0.95	0.987
Sichuan	0.991	0.989	0.977	0.965	0.988	1	0.721	0.928	0.945
Guizhou	0.965	0.948	0.957	0.982	0.98	0.958	0.988	0.959	0.967
Yunnan	1	1	1	1	1	1	1	1	1
Tibet	1	1	1	1	1	1	1	1	1
Shaanxi	0.97	0.993	0.997	1	1	0.984	1	1	0.993
Gansu	1	1	1	1	1	1	1	1	1
Qinghai	0.993	1	0.995	1	1	1	1	1	0.999
Ningxia	1	1	1	1	1	1	1	1	1
Xinjiang	1	1	1	1	1	1	1	1	1
Mean	0.981	0.982	0.983	0.986	0.986	0.983	0.973	0.977	0.981
Efficient DMUs	16	17	18	22	21	19	19	18	18.75

## Appendix A. The Input Slacks for 2011–2018

**Table A1:** The slack of licensed doctors over 2011–2018.

	2011	2012	2013	2014	2015	2016	2017	2018
Beijing	15,988	17,439	16,407	17,250	19,517	19,511	18,934	26,551
Tianjin	5378	5447	5453	5463	6799	7286	9051	9539
Hebei	0	0	0	0	23,487	0	28,552	36,823
Shanxi	23,643	23,135	23,623	23,553	23,805	22,814	22,191	24,189
Inner Mongolia	9645	9935	11,070	10,310	11,528	11,415	10,622	11,218
Liaoning	17,753	17,072	12,322	11,204	12,265	11,939	18,924	23,839
Jilin	11,267	10,947	10,436	9590	11,596	12,557	11,693	13,443
Heilongjiang	15,795	15,283	12,803	13,932	11,368	8533	0	14,340
Shanghai	0	0	0	0	0	0	0	0
Jiangsu	10,900	15,265	13,685	12,893	13,048	12,899	22,654	24,580
Zhejiang	0	0	0	0	0	0	0	0
Anhui	4479	7593	6128	5729	4238	6174	7403	9795
Fujian	0	0	0	0	0	0	0	2752
Jiangxi	1092	1283	355	0	0	0	0	2624
Shandong	0	0	0	0	0	0	0	0
Henan	0	0	0	0	0	1380	2116	0
Hubei	0	797	16	0	0	0	0	0
Hunan	0	0	0	0	0	0	0	0
Guangdong	0	0	0	0	0	0	0	0
Guangxi	0	0	0	0	0	0	0	0
Hainan	1263	1142	976	1015	1061	1051	0	1101
Chongqing	1763	2107	3047	3451	3332	7399	5461	9853
Sichuan	2720	3793	4734	7297	3107	0	44282	13,788
Guizhou	3302	5047	4095	3088	3767	7375	4988	8345
Yunnan	0	0	0	0	0	0	0	0
Tibet	0	0	0	0	0	0	0	0
Shaanxi	6977	6511	6569	6437	5563	8743	0	9614
Gansu	0	0	0	0	0	0	0	0
Qinghai	0	0	0	0	0	0	0	0
Ningxia	291	0	269	538	262	1005	1224	939
Xinjiang	0	0	0	0	0	0	0	0

**Table A2:** The slack of registered nurses over 2011–2018.

	2011	2012	2013	2014	2015	2016	2017	2018
Beijing	22,027	22,244	19,163	21,677	24,009	21,775	15,439	30,828
Tianjin	3608	3899	3552	3960	4922	5251	6280	7529
Hebei	0	0	0	0	1393	0	4084	12,802
Shanxi	24,065	22,495	26,430	26,594	28,948	29,939	34,729	31,797
Inner Mongolia	9370	10,906	12,967	14,740	17,149	17,199	18,373	14,949
Liaoning	23,256	22,359	19,623	18,182	16,119	19,724	23,526	32,817
Jilin	10,402	9667	9820	9890	11,273	15,965	14,024	17,300
Heilongjiang	18,059	18,409	16,669	16,516	14,166	13,564	0	17,891
Shanghai	0	0	0	0	0	0	0	0
Jiangsu	19,351	25,487	23,651	24,405	23,702	22,970	33,120	38,559
Zhejiang	0	0	0	0	0	0	0	0
Anhui	9038	12,332	11,271	10,592	11,090	11,779	8619	16,227
Fujian	0	0	0	0	0	0	0	11,553
Jiangxi	3790	4167	4023	0	0	0	0	10,323
Shandong	0	0	0	0	0	0	0	0
Henan	0	0	0	0	0	2477	3765	0
Hubei	0	637	29	0	0	0	0	0
Hunan	0	0	0	0	0	0	0	0
Guangdong	0	0	0	0	0	0	0	0
Guangxi	0	0	0	0	0	0	0	0
Hainan	3420	4135	4361	4979	5241	4927	0	6026
Chongqing	3048	4721	5662	6536	7324	14,720	12,014	19,390
Sichuan	4316	6328	8163	12,977	10,418	0	88,857	27,495
Guizhou	6113	8345	7092	5826	7128	14,292	9681	16,525
Yunnan	0	0	0	0	0	0	0	0
Tibet	0	0	0	0	0	0	0	0
Shaanxi	13,458	14,421	16,486	16,009	14,618	18,516	0	23,218
Gansu	0	0	0	0	0	0	0	0
Qinghai	0	0	0	0	0	0	0	0
Ningxia	1988	0	1368	873	558	1391	3671	3279
Xinjiang	0	0	0	0	0	0	0	0

**Table A3:** The slack of other technical staff over 2011–2018.

	2011	2012	2013	2014	2015	2016	2017	2018
Beijing	4297	4414	4585	5249	5378	5071	4227	6749
Tianjin	3357	3650	3660	3704	3491	3285	3290	3059
Hebei	0	0	0	0	136	0	927	1796
Shanxi	3266	3026	3607	3297	3387	4086	3861	4081
Inner Mongolia	1068	1367	1906	2575	2757	2927	3134	3361
Liaoning	3119	3612	3201	3071	2967	4196	4401	4808
Jilin	3777	3937	4219	3971	4427	4896	4315	5256
Heilongjiang	5106	5511	5370	5340	4872	4930	0	5639
Shanghai	0	0	0	0	0	0	0	0
Jiangsu	2149	2636	2259	2117	2158	1926	3407	4094
Zhejiang	0	0	0	0	0	0	0	0
Anhui	782	1204	1022	927	686	987	724	1350
Fujian	0	0	0	0	0	0	0	203
Jiangxi	91	204	52	0	0	0	0	17
Shandong	0	0	0	0	0	0	0	0
Henan	0	0	0	0	0	1423	2369	0
Hubei	0	122	114	0	0	0	0	0
Hunan	0	0	0	0	0	0	0	0
Guangdong	0	0	0	0	0	0	0	0
Guangxi	0	0	0	0	0	0	0	0
Hainan	353	554	655	744	855	964	0	783
Chongqing	994	1266	1815	2081	2503	4284	3986	3921
Sichuan	2985	3278	4017	5763	3725	0	12,426	5308
Guizhou	1095	1770	1371	871	1719	3095	2683	2964
Yunnan	0	0	0	0	0	0	0	0
Tibet	0	0	0	0	0	0	0	0
Shaanxi	5009	5322	5929	6333	6635	7603	0	9077
Gansu	0	0	0	0	0	0	0	0
Qinghai	0	0	0	0	0	0	0	0
Ningxia	52	0	44	90	7	157	361	256
Xinjiang	0	0	0	0	0	0	0	0

**Table A4:** The slack of beds over 2011–2018.

	2011	2012	2013	2014	2015	2016	2017	2018
Beijing	7905	7454	3528	6888	4078	372	17,543	9866
Tianjin	1257	4549	5876	7082	8128	7153	9681	9141
Hebei	0	0	0	0	8614	0	25,582	42,245
Shanxi	42,944	40,256	42,434	42,708	49,704	49,898	58,443	53,914
Inner Mongolia	17,090	20,364	23,937	26,877	30,804	29,903	32,266	26,288
Liaoning	44,460	43,789	38,802	39,751	33,600	51,487	67,758	76,685
Jilin	21,486	20,162	21,162	21,180	23,816	33,927	28,612	34,953
Heilongjiang	34,838	36,532	33,541	34,281	30,014	28,680	0	58,769
Shanghai	0	0	0	0	0	0	0	0
Jiangsu	43,864	56,405	60,558	62,028	54,313	59,537	81,385	84,580
Zhejiang	0	0	0	0	0	0	0	0
Anhui	14,126	22,141	20,333	19,187	14,475	21,021	15,582	29,533
Fujian	0	0	0	0	0	0	0	4246
Jiangxi	1569	3885	1137	0	0	0	0	456
Shandong	0	0	0	0	0	0	0	0
Henan	0	0	0	0	0	33031	46339	0
Hubei	0	1146	840	0	0	0	0	0
Hunan	0	0	0	0	0	0	0	0
Guangdong	0	0	0	0	0	0	0	0
Guangxi	0	0	0	0	0	0	0	0
Hainan	847	1860	292	564	971	792	0	69
Chongqing	15,108	21,897	27,185	26,650	35,344	29,069	46,442	38,057
Sichuan	10,876	38,725	42,204	52,037	40,443	0	238,709	86,017
Guizhou	10,696	20,792	25,462	39,597	41,320	29,004	48,153	32,432
Yunnan	0	0	0	0	0	0	0	0
Tibet	0	0	0	0	0	0	0	0
Shaanxi	21,091	20,591	22,086	33,954	32,117	30,009	0	32,417
Gansu	0	0	0	0	0	0	0	0
Qinghai	0	0	0	0	0	0	0	0
Ningxia	878	0	1786	1638	122	2505	3682	2512
Xinjiang	0	0	0	0	0	0	0	0
